# Effect of Ileal Transposition (IT) on Angiopoietin-Like Protein-8 (ANGPTL8) and Pentraxin (PTX3) Plasma Level in Sprague-Dawley Rats Fed High-Fat Diet (HFD)

**DOI:** 10.1155/2021/6699923

**Published:** 2021-05-05

**Authors:** Tomasz Sawczyn, Dominika Stygar, Katarzyna Nabrdalik, Michał Kukla, Oliwia Masri, Łukasz Magrowski, Wojciech Karcz, Jerzy Jochem

**Affiliations:** ^1^Department of Physiology, Faculty of Medical Sciences, Zabrze, Medical University of Silesia, Katowice, Poland; ^2^Department of Internal Medicine, Diabetology and Nephrology, Zabrze, Medical University of Silesia, Katowice, Poland; ^3^Department of Internal Medicine and Geriatrics, Jagiellonian University Medical College, Krakow, Poland; ^4^Department of General, Visceral, Transplantation and Vascular Surgery, Hospital of the Ludwig Maximilian University, Munich, Germany

## Abstract

**Background:**

Metabolic surgery procedures are designed not only for sustained weight loss but also for achieving positive metabolic changes, including improved glucose tolerance and insulin sensitivity, along with an increase in energy expenditure. Based on recent findings, the present study focuses on the relationship between the effects of ileal transposition (IT), high-fat diet (HFD), and selected markers of lipid metabolism and inflammation.

**Methods:**

Forty-eight male rats were divided into two groups: HFD and control diet (CD) fed rats. After eight weeks, animals in each group were randomly assigned to two types of surgery: IT and SHAM. Thereafter, fifty percent of the animals in the HFD and CD groups had their diets changed, while the remaining half maintained their presurgery diets. Eight weeks after surgery, plasma levels of ANGPTL8, PTX3, leptin, and adiponectin were assessed.

**Results:**

The IT group pre- and postoperatively maintained on the HFD showed higher ANGPTL8 level compared to SHAM operated animals (*p*=0.0041). The effect of IT on PTX3 level in the group pre- and postoperatively maintained on a CD was not significant, and there were no differences compared to SHAM. Only the postoperative diet change to HFD increased PTX3 level in the IT operated animals (*p*=0.0002). The IT group had increased plasma adiponectin (*p*=0.026) and leptin (*p*=0.0027) levels after dietary change to HFD compared to IT rats fed CD.

**Conclusions:**

This study indicates that the outcomes of metabolic surgery can be greatly modified by HFD. The effects of the IT procedure in this experiment are ambiguous and do not provide a clear answer as to whether or not they are beneficial.

## 1. Background

The conclusion that IT is a powerful metabolic regulator has already been reproduced in many studies [1–3]. Nowadays, the remarkable effects of metabolic surgery are considered not purely as an effective way to weight loss, but also as a therapeutic modality in type 2 diabetes mellitus (T2DM) [4]. Furthermore, it is being attempted to use it as a means to regulate obesity-induced inflammation as well as metabolic and hormonal activity of adipose tissue [5, 6]. Recently, several triggering factors have been discovered that may strongly modify the beneficial effects of metabolic surgery [7, 8]. The type of diet seems to carry out a major role in modulating the effects of metabolic surgery, especially considering that dietary interventions after surgery are crucial and still the most difficult to implement, especially in the long term [9].

The role of ANGPTL8 in lipid metabolism has already been demonstrated and the circulating level of this protein is associated with intrinsic energy intake and expenditure [10, 11]. The ANGPTL proteins (ANGPTL3, ANGPTL4, and ANGPTL8) are a family of differentially regulated lipoprotein lipase inhibitors primarily expressed in white adipose tissue (WAT) [12, 13]. In the postprandial period, elevated circulating insulin level increases ANGPTL8 expression, leading to an increase in lipoprotein lipase activity and lipid uptake in adipose tissue and a simultaneous decrease in skeletal muscle. Vatner et al. [14] postulated that pharmacological modulation of endogenous lipoprotein lipase regulating factors such as ANGPTL8 may represent a novel approach to prevent or treat insulin resistance and NAFLD. These prior reports prompted us to study the plasma level of native ANGPTL8 as an indicator of metabolic changes after IT surgery in diet-induced obesity.

Obesity is a systemic inflammatory pathological state associated with increased levels of numerous inflammatory markers including C-reactive protein (CRP), IL-6, TNF-*α*, and some adipokines associated with inflammatory factors such as RBP4 or chemerin [15–17]. Metabolic surgery is recognized to improve various markers of inflammation and ameliorates chronic inflammatory status [18–21]. However, it is important to point out that some studies do not support these conclusions and show no change or even an increase in serum TNF-*α* levels following metabolic surgery [22–24]. Pentraxin 3 (PTX3) serves a role in protection against infection, inflammation control, and matrix deposition and has been proven as a biomarker of inflammatory status. Immune cells, such as monocytes and neutrophils, serve as major sources of PTX3. Some cross-sectional studies demonstrated that adipocytes express PTX3 [25, 26]. Tonial et al. [27] found that obese patients had lower plasma levels of PTX3 compared to control patients, and the levels were restored to the physiological norm following bariatric surgery. The authors postulated that this effect might be associated with weight loss. However, animal studies showed that PTX3 deficiency reduced metabolic inflammation and prevented weight gain in mice fed HFD [28]. Therefore, it is worth investigating how plasma PTX3 level will change after IT surgery, which exerts minor effect on weight loss, and how HFD will affect this parameter.

Adipokines secreted from adipose tissue show pleiotropic activity and are considered both pro- and anti-inflammatory factors. They are also involved in the regulation of glucose homeostasis [29]. To investigate anti-inflammatory factors associated with adipocyte activity and HFD, we selected two adipokines whose metabolic activity has been studied most extensively: adiponectin and leptin. Adiponectin is mainly secreted by adipocytes as an anti-inflammatory agent that also improves insulin sensitivity. The majority of studies demonstrated a significant increase in circulating adiponectin levels for at least three months after bariatric surgery [15, 23, 24], while leptin plasma concentrations decrease after bariatric surgery, regardless of time points of follow-up or type of surgical procedures [30, 31]. Adiponectin seems to be a promising intervention for the treatment of metabolic disorders; thus its high plasma levels could be considered as a benefit of IT. Leptin suppresses food intake, increases energy expenditure, and promotes weight loss. Genetic deficiency of leptin or leptin receptors results in hyperphagia and severe obesity in both rodents and humans [32–34]. However, circulating leptin levels increase under most obesity conditions, and diet-induced obesity is associated with leptin resistance leading to excessive food intake and body weight gain [35].

This research aimed to investigate the influence of pre- and postoperative HFD on IT effects and assessing plasma levels of selected indicators that could be useful as potential biomarkers for assessing the physiological state of patients undergoing IT surgery or other bariatric/metabolic procedures.

## 2. Materials and Methods

### 2.1. Animals and Experimental Design

Animal care and handling were performed following the principles of the 3Rs (Replacement, Reduction, and Refinement), and all experimental procedures received the approval from the Local Ethical Committee for Experiments on Animals in Medical University of Silesia in Katowice (No.146/2016). Eight weeks old, male Sprague-Dawley rats, initially weighing between 250 and 275g, were purchased from the Centre for Experimental Medicine, Medical University of Silesia, and were housed in plastic cages under controlled conditions (ambient temperature, 23°C ± 1°C; 12 h light/dark cycle). The number of rats was kept as low as possible considering the “3Rs” for the humane treatment of animals [36].

**Figure 1:**
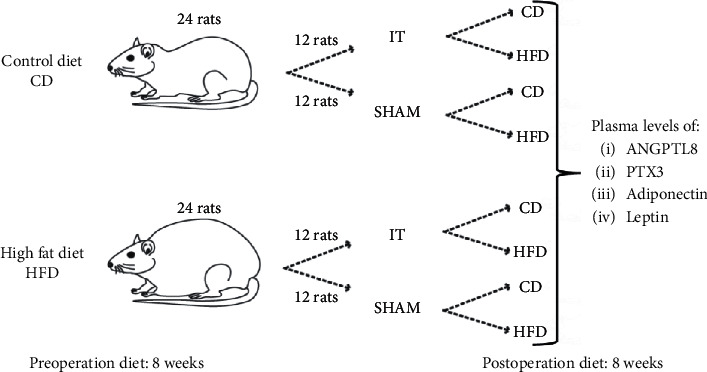
Scheme of experimental groups.

Forty-eight male rats were used in the experiment. The male rats are characterized by a more stable hormonal milieu and body mass fluctuations. After a two-week acclimatization phase, the rats were fed ad libitum HFD (*n* = 24) or control diet (CD) (*n* = 24) for 8 weeks (Figure 1). After 8 weeks, the final preoperative oral glucose tolerance test (OGTT) was performed (data not presented). Experimental HFD supports the development of the metabolic syndrome and glucose intolerance. Thus, the endpoint of the presurgery diet was to achieve significantly higher body weight and lower glucose tolerance (measured by the OGTT) in the HFD group compared to the CD fed animals.

**Figure 2:**
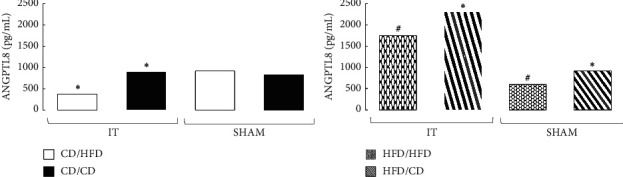
Median of ANGPTL8 plasma concentration in four diet groups according to operation type. Abbreviations: IT—ileal transposition, SHAM—sham operated animals, CD—control diet, and HFD—high-fat diet. Preoperative diet/postoperative diet: CD/HFD, CD/CD, HFD/HFD, HFD/CD (group size: *n* = 6, except IT HFD/HFD: *n* = 5). Sign ^*∗*^ or ^#^ denotes significant difference between median values; Kruskal–Wallis test, *p* < 0.05. Quartile deviation (Q25 and Q75) available in Supplementary Materials.

The HFD fed and CD fed rats were randomly assigned to two types of surgery: IT and SHAM. On the third day after surgery, the diet was changed in 50% of the animals in each group, while the remaining animals received the same type of diet as presurgery (Figure 2). The rats were housed on postoperative diets for 8 weeks. The rat's body weight was recorded every two days before surgery, daily for 7 days after surgery, and every two days for the remaining period. No morbidities occurred in all groups of rats during the experiment. Mortality related to surgery was 2% (one rat IT HFD/HFD group).

### 2.2. Diet Composition

All diets were purchased from Ssniff Spezialdiäten GmbH (Soest, Germany). Diet composition, expressed as a percentage of metabolisable energy (ME), was the same as in our previous study [37]: control diet (referred to as CD in the following) (D12450 B I mod.; Ssniff): 9% from fat, 26% from protein, and 65% from carbohydrates (ME = 3,77 kcal/g); HFD (D12492 II mod.; Ssniff): 60% from fat, 19% from protein, and 21% from carbohydrates (ME = 5,75 kcal/g).

### 2.3. Metabolic Surgery Procedures

**Figure 3:**
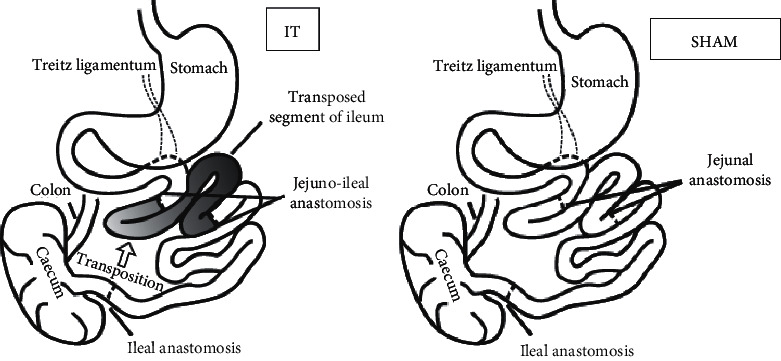
Schematic illustration of IT and SHAM surgery respectively.

After fasting for 24 hours but with unrestricted access to tap water, rats underwent IT or SHAM surgery (Figure 3). All rats were operated under 2–3% isoflurane anesthesia and an oxygen flow of 2 L/min under spontaneous breathing and xylazine premedication (5 mg/kg of body weight). After a midline incision of 4–5 cm to gain abdominal access, the ileocecal valve (Bauhin's valve) was identified. The positions of the two transections necessary to isolate the ileal segment were determined relative to the Bauhin valve for 50% of the distal ileum. The first anastomosis was then formed as an end-to-end ileostomy to restore ileal continuity, excluding the transposed segment. All anastomoses were performed as interrupted end-to-end extramucosal anastomoses using PDS 6/0 (Ethicon, Blue Ash, OH). The Treitz ligament was then identified and the jejunum was divided 5 cm aborally. The ileal segment was then isoperistaltically interposed to create two end-to-end anastomoses. For SHAM surgery, transections were made at all three corresponding positions. The anastomoses were then formed accordingly without IT. Fascia and skin closure were conducted as continuous suture using Monocryl 4/0 and Vicryl 4/0 (Ethicon, Blue Ash, OH). Postoperative analgesia was ensured via subcutaneous carprofen (Rycarfa 50 mg/ml, Krka) injection (4 mg/kg). After the operation, animals were maintained on a liquid diet (Nutrison, Nutricia, Poland) for 24 hours. Following this period, when the rats did not present any symptoms of postoperative complications, they were randomly assigned to groups maintained on varied postoperative diets [37].

### 2.4. Blood Sampling

Before the blood sampling, rats ware fasted for 12 h. The blood required for the determination of glucose, peptides, and insulin plasma levels was collected. The volume of 400 *μ*L of whole blood was collected via the tail vein cannula, using tubes containing 10 *μ*L EDTA (Sigma-Aldrich, St. Louis, MO). A 4 *μ*L protease inhibitor was added to the tube prior to blood collection to inhibit peptide degradation. After centrifugation at 4000 rpm for 10 min at 4°C, plasma samples were collected and snap-frozen in liquid nitrogen and stored at minus 80°C until analysis.

### 2.5. Hepatokines and Adipokines Plasma Assessment

Plasma levels of ANGPTL8, PTX3, adiponectin, and leptin were measured weekly during the postoperative period. Peptides plasma concentrations were assessed in duplicate by the immunoenzymatic method with the commercially available ELISA kits (USCN Life Science Inc., USA) using rat peptides as a standard.

### 2.6. Tissue Harvesting and Liver Histology

Liver tissue harvesting was the terminal procedure. All rats before being sacrificed underwent 2–3% isoflurane anesthesia and oxygen flow of 2 L/min under spontaneous breathing and xylazine premedication (5 mg/kg of body weight). Before the left median lobe of the liver was explanted, the liver was perfused with PBS. The liver lobes of each rat were divided into six to seven sections for further slide preparation. A procedure consisting of a combination of tail bleeding and cardiac puncture allowed the controlled collection of predetermined amounts of blood from rats for further biochemical analysis. After blood sampling, the animal was bleeding out.

Liver sections were fixed in paraformaldehyde and embedded in paraffin. The paraffin sections were stained with hematoxylin-eosin (HE). One slide from each section was chosen for histopathological evaluation. Histopathological features were evaluated according to Scheuer's classification with grading of inflammatory activity (G1-G4) and fibrosis (F1–F4) [38]. Steatosis was graded as follows: *S*1 <33%, S2 33–66%, and S3 more than 66% of hepatocytes affected [39]—ballooning: 0, none, 1, rare or few, 2, moderate, and 3, many.

### 2.7. Statistical Procedures

All statistical procedures were performed as in our earlier study [37]. Briefly, the concentration of plasma hepatokines and adipokines did not fulfil the criteria of normal distribution (Shapiro–Wilk test). Consequently, the Kruskal–Wallis test (*p* < 0.05) was used for group comparison and the results were expressed as a median and quartile deviation (25^th^ and 75^th^ percentiles). The Wilcoxon signed-rank test was used for two related samples' comparison (fasting and postglucose load states). Other measured parameters were assessed with the two-way analysis of variance (ANOVA). All survived rats were included in the statistical analyses. The Dixon *Q* test was performed to detect significant outliers. The level of statistical significance was set at *p* < 0.05. Statistical analyses were performed using Statistica 10.0.

## 3. Results

### 3.1. ANGPTL8 Plasma Level

The effect of IT on the ANGPTL8 level did not occur in the groups preoperatively maintained only on CD. Only the diet change from CD to HFD resulted in a significant decrease in the ANGPTL8 plasma level (*p*=0.0038). The effect of IT on the circulating ANGPTL8 level was more pronounced in the rats fed HFD preoperatively. The IT group fed with HFD preoperatively and postoperatively had higher ANGPTL8 levels compared to the SHAM operated animals (*p*=0.0041). A similar effect was observed in the IT operated HFD/CD group compared to SHAM (*p*=0.0047). In both cases, the increase in ANGPTL8 concentration was 2.5 times higher in the IT groups than in the SHAM rats, and a dietary change seems to support this trend (Figure 2).

### 3.2. Pentraxin 3 (PTX3) Plasma Level

**Figure 4:**
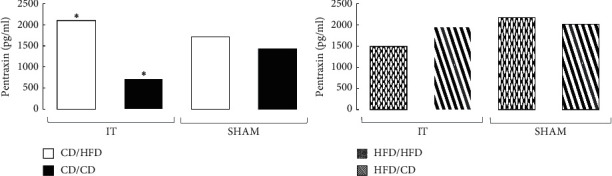
Median of PTX3 plasma concentration in four diet groups according to operation type. Abbreviations: IT—ileal transposition, SHAM—sham operated animals, CD—control diet, and HFD—high-fat diet. Preoperative diet/postoperative diet: CD/HFD, CD/CD, HFD/HFD, HFD/CD (group size: *n* = 6, except IT HFD/HFD: *n* = 5). Sign *∗* denotes significant difference between median values; Kruskal–Wallis test, *p* < 0.05. Quartile deviation (Q25 and Q75) available in Supplementary Materials.

The effect of IT on PTX3 level in the group maintained on CD pre- and postoperatively was not significant, and there were no differences compared with SHAM. Only the postoperative diet change to HFD increased PTX3 level in IT operated animals (*p*=0.0002). In the groups preoperatively fed HFD, the effect of IT and postoperative diet on plasma PTX3 levels was barely noticeable and statistically insignificant (Figure 4).

### 3.3. Leptin Plasma Level

**Figure 5:**
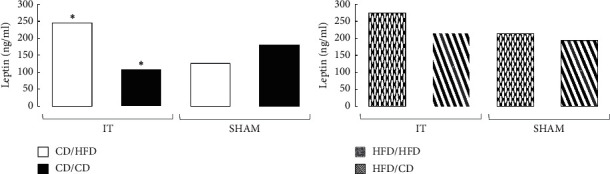
Median of leptin plasma concentration in four diet groups according to operation type. Abbreviations: IT—ileal transposition, SHAM—sham operated animals, CD—control diet, and HFD—high-fat diet. Preoperative diet/postoperative diet: CD/HFD, CD/CD, HFD/HFD, HFD/CD (group size: *n* = 6, except IT HFD/HFD: *n* = 5). Sign *∗* denotes significant difference between median values; Kruskal–Wallis test, *p* < 0.05. Quartile deviation (Q25 and Q75) available in Supplementary Materials.

IT had no effect on the leptin plasma level in rats preoperatively fed HFD. Only changing the diet to HFD in IT rats increased the plasma leptin level (*p*=0.0027). In general, plasma leptin levels were higher in all HFD groups compared to CD fed rats, but the difference did not reach statistical significance (Figure 5).

### 3.4. Adiponectin Plasma Level

**Figure 6:**
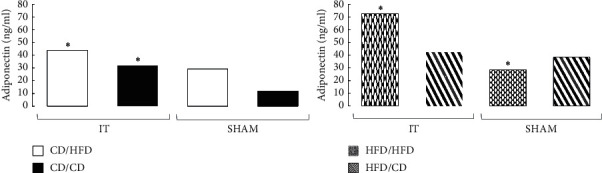
Median of adiponectin plasma concentration in four diet groups according to operation type. Abbreviations: IT—ileal transposition, SHAM—sham operated animals, CD—control diet, and HFD—high-fat diet. Preoperative diet/postoperative diet: CD/HFD, CD/CD, HFD/HFD, HFD/CD (group size: *n* = 6, except IT HFD/HFD: *n* = 5). Sign *∗* denotes significant difference between median values; Kruskal–Wallis test, *p* < 0.05. Quartile deviation (Q25 and Q75) available in Supplementary Materials.

Among the rats preoperatively maintained on CD, IT surgery without a diet change resulted in a minor increase in adiponectin plasma level compared to SHAM, but did not achieve statistical significance. The IT group after the diet change to HFD was characterized by an increased adiponectin level compared to IT rats fed CD (*p*=0.026), without significant differences compared to SHAM. Meanwhile, obese rats fed only HFD during the experiment revealed after IT a twofold higher adiponectin level than SHAM operated rats (*p*=0.007). The IT operation did not affect the adiponectin level in the group of obese rats whose diet was changed to CD (Figure 6; Supplement Table 4).

### 3.5. Histopathology of the Liver

**Figure 7:**
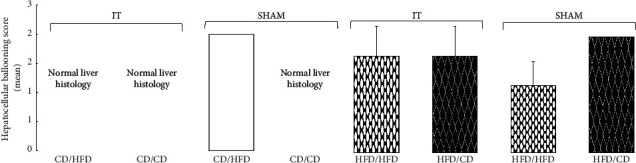
Liver histopathology. Distribution of normal and degenerated hepatocytes (ballooning degeneration) in four diet groups according to the operation type (IT and SHAM). Abbreviations: IT—ileal transposition, SHAM—sham operated animals, CD—control diet, and HFD—high-fat diet. Preoperative diet/postoperative diet: CD/HFD, CD/CD, HFD/HFD, HFD/CD. Data are presented as the means ± SEM.

Fibrosis and inflammatory activity were not observed in any of the groups. IT operated rats from the CD/HFD and CD/CD groups had normal liver histology, similar to SHAM operated rats in the CD/CD group. Steatosis (grade S1) was found only in 40% of the SHAM CD/HFD group. Mild ballooning of hepatocytes was found in 83% of SHAM operated rats from the HFD/HFD group. Hepatocellular ballooning (grade 2) was disclosed in IT operated rats from the HFD/HFD and HFD/CD groups and in SHAM operated rats from the HFD/CD group (Figure 7).

## 4. Discussion

### 4.1. ANGPTL8

In the present study, we found that HFD after IT surgery affected plasma ANGPTL8 level. Dietary change from CD to HFD in the IT group resulted in a significant decrease in the ANGPTL8 plasma level; however, this effect was not reproduced in the SHAM groups. These results seem to be in contrast with the findings of the studies on the effect of HFD on plasma ANGPTL8 levels in mice, where HFD administration induced ANGPTL8 mRNA production in the liver and white and brown adipose tissues [12, 40, 41]. However, it should be taken into account that this group was fed with CD until operation and HFD was implemented after IT. The diet change to HFD does not lead to significant increase in body weight or development of glucose intolerance in rats, as we have shown in our previous study [37]. Interestingly, our results are more consistent with those obtained by Franck et al. [42] in a study on caloric intake-related genes in human adipocytes. In that study, ANGPTL8 mRNA level decreased in the lean group after overfeeding and increased after ordinary food reintroduction in the obese group, which had previously been given the low-calorie diet. In our experiment, IT operated rats pre- and postoperatively fed HFD had lower ANGPTL8 plasma level compared to rats postoperatively fed CD. A similar tendency was visible in the SHAM groups. Despite the lack of statistical significance, a comparable pattern of variation in plasma ANGPTL8 level after diet change in the IT and SHAM groups attracts attention. Being aware that human and animal models are relatively far apart, and that mRNA is regulated and not necessarily associated with high protein presence in plasma, we can only conclude that it is related to a type of diet.

In addition, we found that metabolic surgery compared to SHAM had no influence on ANGTPL8 plasma level in CD/CD and CD/HFD rats, whereas IT strongly increased ANGTPL8 in rats fed HFD preoperatively, regardless of the dietary change compared. Studies in rodents focus mainly on the influence of diet on circulating ANGPTL8 concentrations. In this study, we have shown for the first time that IT causes an increase in the ANGPTL8 plasma level in rats with diet-induced obesity. There are very few studies regarding the level of circulating ANGPTL8 after metabolic surgery. Previous studies on human plasma levels of ANGPTL8 reported a decrease in circulating levels of ANGPTL8 in morbidly obese individuals and an increase after bariatric surgery [43, 44]. In parallel, another study showed that serum ANGPTL8 concentrations were elevated in obese subjects after surgically induced weight loss, but not after conventional dietary treatment [45]. Studies in rodents conducted by Vatner et al. [14] explored the role of ANGPTL8 pertaining to its regulation of lipoprotein lipase in adipose tissue. The authors indicated the potential key role of ANGPTL8 as a regulatory factor for lipid metabolism by using a second-generation antisense oligonucleotide (ASO) against this protein in adult high-fat diet fed rodents. In that study, the ANGPTL8 ASO increased lipid uptake from chylomicrons into adipose tissue in rats fed HFD. Lipid uptake from subcutaneous adipose tissue was also increased in rats fed normal chow. Based on the available literature data regarding the concentration of circulating ANGPTL8 in obesity and T2DM, such results suggest that this protein is associated with intrinsic energy intake and expenditure. A high ANGPTL8 plasma level seems to be considered an advantage of the IT surgery, as observed in groups preoperatively fed HFD. However, our recent study showed no significant differences in HOMA-IR and insulin plasma levels between the IT and SHAM operated animals and the different diets [37]. ANGPTL8 is primarily expressed in the liver and adipose tissue, from where it is secreted into the bloodstream. It can be assumed that the second mentioned source is the main reason for the higher plasma levels of ANGPTL8 in HFD/HFD and HFD/CD, IT operated rats than in those which have undergone SHAM surgery. A poor histological condition of the liver with hepatocellular ballooning occurring in all abovementioned groups of rats seems to confirm this claim. In our previous research, we also pointed out that IT surgery significantly alters the adipose tissue metabolism, in both its enzymatic processes and secretory capacity [15].

### 4.2. Pentraxin 3

Obesity, as a consequence of nutrient excess and loss of metabolic homeostasis, contributes to a chronic inflammatory state that increases systemic proinflammatory mediators. Metabolic surgery is known to improve this chronic low-grade inflammation. We have shown in our earlier study that IT surgery leads to a decrease in plasma levels of chemerin and adipokines associated with inflammatory factors, including C-reactive protein (CRP), IL-6, and tumor necrosis factor (TNF) [37, 45].

PTX3 has been reported to be associated with adipose tissue and obesity in a genetic mouse model of obesity [46]. Human studies have suggested that obese individuals have elevated plasma PTX3 levels [47] and increased expression of PTX3 in visceral adipose tissue, which have been linked with cardiovascular diseases. However, literature data show some discrepancies. A study performed on nine healthy individuals indicated that a decrease in body fat mass was associated with increased PTX3 plasma concentrations [48]. Other studies demonstrate an inverse relationship between body mass and systemic PTX3 concentrations [49, 50]. In our study, neither IT surgery nor diet change to CD resulted in a significant change in plasma PTX3 levels. Although not statistically significant, there was a minor decrease in PTX3 plasma levels in the IT operated rats fed HFD constantly. However, this group was characterized by significantly higher PTX3 plasma levels than the IT group maintained during the experiment only on CD. This observation suggests that HFD induces PTX3 expression. The increase of plasma PTX3 level following dietary change to HFD in IT operated rats also seems to support the above hypothesis. This change in diet led to four times higher PTX3 levels compared to the CD fed group. It is also important to note that SHAM operated animals had almost the same level of PTX3 as IT rats in the corresponding diet groups. It is worth mentioning that IT operated rats fed HFD postoperatively had higher HOMA-IR levels than rats fed CD. In addition, in originally normoglycemic rats that underwent IT surgery and changed to HFD, the incretin effect was significantly impaired or did not occur at all [37]. Bonacina et al. [28] received increased PTX3 levels and impaired glucose tolerance and insulin sensitivity after feeding mice with HFD for 10 and later 20 weeks. Current results show that dietary conversion to HFD causes a significant increase in plasma PTX3 level, despite the IT metabolic effect that occurs in rats fed CD. Data obtained by Witshap et al. [51] support an inverse association between circulating PTX3 and anthropometric measures such as adiposity. The authors also report a positive association between circulating PTX3 and adiponectin, suggesting that PTX3 has both anti-inflammatory and antidiabetic effects. It is important to mention that not only adiponectin is located in the same chromosomal region (3q) as PTX3, but is also homologous to its ligand C1q [52].

### 4.3. Adiponectin

In contrast to the discrepancies discussed above regarding the role of PTX3, the activity of adiponectin in obesity has been studied more broadly. Adiponectin exhibits pleiotropic autocrine and paracrine functions, including its insulin-sensitizing and anti-inflammatory effects. It also prevents ectopic lipid accumulation by enhancing lipid storage in adipocytes and reducing hepatic gluconeogenesis [53, 54]. Adiponectin appears to offer a promising intervention for the treatment of metabolic disorders; therefore its high plasma level could be considered a benefit of IT. In our study, IT surgery significantly increased plasma adiponectin level in rats fed HFD constantly, compared to the SHAM group. However, switching the diet from HFD to CD attenuated this effect, resulting in nearly equal adiponectin plasma levels in both IT and SHAM operated rats. In this case, HFD seems to support adiponectin secretion after IT operation. The same tendency can be observed in the control group, in which plasma adiponectin levels increased significantly after dietary change from CD to HFD in IT operated rats, compared with IT operated rats, which were fed constant CD during the experiment. Based on our data alone, it is difficult to explain the described changes. We put forward the hypothesis that higher content of fatty acids in HFD could influence metabolic flux. Higher fat supply and lipids secreted from adipocytes serve as signaling molecules that regulate energy metabolism. Furthermore, our recent study showed that glucose tolerance assessed by OGTT revealed comparable AUC between IT and SHAM operated animals in both CD and HFD groups [37] (Supplement Figure 1). Only IT and SHAM operated rats maintained on CD after surgery had the highest fasting plasma glucose level among all groups. This suggests that the intake of fat or carbohydrate after IT surgery significantly affects the energy metabolism. We believe that further studies are needed to assess the direction of these changes.

### 4.4. Leptin

Circulating leptin levels are proportional to fat mass, and leptin receptors are abundantly expressed in many tissues, including adipocytes [55]. It was therefore not surprising that the highest plasma leptin level was found in rats maintained only on HFD. Both IT surgery and diet change to CD did not result in significant changes in plasma leptin level. In most studies, it was observed that a significant decrease in body weight, accompanied by reduced caloric intake and improved food efficiency after metabolic surgery (i.e., Roux-en-Y Gastric Bypass: RYGB), led to decreasing leptin plasma level. Our data seem to be consistent with the above. As we indicated in our previous experiment, IT operated rats did not show significant weight loss, and caloric intake was higher in this group than in SHAM operated animals, so that the leptin plasma level did not change. Although there was no significant effect of IT surgery on leptin plasma levels in the HFD/HFD and HFD/CD groups, a significant dietary effect was found in the IT operated CD/HFD group. In this group, the leptin plasma level increased significantly compared to IT operated CD/CD rats. These data show that HFD has a strong effect on leptin plasma level, even when the opposite metabolic stimulus is given, which could be the effect of IT surgery.

## 5. Conclusions

This study suggests that the beneficial effects of IT surgery can be modified through the HFD. It has been pointed out several times that IT is a strong metabolic regulator, and as we have shown in our previous studies, its effects are time-dependent and continue to fade after surgery.

## Data Availability

All the data are included in the manuscript.

## Abbreviations

IT: Ileal transposition
HFD: High-fat diet
CD: Control diet
SHAM: Sham operated animals
OGTT: Oral glucose tolerance test
T2DM: Type 2 diabetes mellitus
NAFLD: Nonalcoholic ratty liver disease
ANGPTL8: Angiopoietin-like protein-8
PTX3: Pentraxin
RBP4: Retinol binding protein
CRP: C-reactive protein.

## Supplementary Materials

Supplementary Materials Table 1: Median (M) and quartile deviation (Q25 and Q75; 25th and 75th percentiles, respectively) of ANGPTL8 plasma concentration in four diet groups according to operation type. *∗* or # denotes significant difference between median values; Kruskal–Wallis test, *p* < 0.05 (group size: *n* = 6, except IT HFD/HFD: *n* = 5). IT—ileal transposition, SHAM—sham operated animals, CD—control diet, and HFD—high-fat diet. Preoperative diet/postoperative diet: CD/HFD, CD/CD, HFD/HFD, HFD/CD. Table 2: Median (M) and quartile deviation (Q25 and Q75; 25th and 75th percentiles, respectively) of PTX3 plasma concentration in four diet groups according to operation type. *∗* denotes significant difference between median values; Kruskal–Wallis test, *p* < 0.05 (group size: *n* = 6, except IT HFD/HFD: *n* = 5).IT—ileal transposition, SHAM—sham operated animals, CD—control diet, and HFD—high-fat diet. Preoperative diet/postoperative diet: CD/HFD, CD/CD, HFD/HFD, HFD/CD. Table 3: Median (M) and quartile deviation (Q25 and Q75; 25th and 75th percentiles, respectively) of leptin plasma concentration in four diet groups according to operation type. *∗* or # denotes significant difference between median values; Kruskal–Wallis test, *p* < 0.05(group size: *n* = 6, except IT HFD/HFD: *n* = 5). IT—ileal transposition, SHAM—sham operated animals, CD—control diet, and HFD—high-fat diet. Preoperative diet/postoperative diet: CD/HFD, CD/CD, HFD/HFD, HFD/CD. Table 4. Median (M) and quartile deviation (Q25 and Q75; 25th and 75th percentiles, respectively) of adiponectin plasma concentration in four diet groups according to operation type. *∗* or # denotes significant difference between median values; Kruskal–Wallis test, *p* < 0.05(group size: *n* = 6, except IT HFD/HFD: *n* =  5).IT—ileal transposition, SHAM—sham operated animals, CD—control diet, and HFD—high-fat diet. Preoperative diet/postoperative diet: CD/HFD, CD/CD, HFD/HFD, HFD/CD. Supplement. Fig. 1: Postoperatively glucose tolerance profile. (A) Area under curve of blood glucose profiles of OGTT. *∗* denotes significant difference between SHAM CD/CD and SHAM CD/HFD groups. # denotes significant difference between SHAM HFD/HFD and SHAM CD/HFD. (B) OGTT curves in four groups of pre-/postoperative diet according to the operation type IT or SHAM surgery. *∗* denotes significant difference between fasting state IT CD/CD and SHAM CD/CD # denotes significant difference between IT CD/HFD and SHAM CD/HFD, 30 min of OGTT. † denotes significant difference between IT CD/HFD and SHAM CD/HFD, 60 min of OGTT. ‡ denotes significant difference between SHAM HFD/HFD and SHAM CD/HFD. *p* < 0.05 by Tuckey's test. Preoperative diet/postoperative diet: CD/HFD, CD/CD, HFD/HFD, HFD/CD. Data are expressed as means ± SD, *n* = 6. *p* < 0.05 by Tukey's test. Supplement. Fig. 2: Weight changes during the experiment. (A) Weight after 8 weeks of HFD and CD; *∗* denotes significant difference between groups. (B) Variability of weight fluctuations in five time periods after the operation; *∗* denotes significant difference between IT CD/CD and SHAM CD/CD groups; # denotes significant difference between SHAM CD/CD and SHAM CD/HFD groups; † denotes significant difference between IT HFD/HFD and SHAM HFD/HFD groups. (C) Body weights' changes during 8 weeks after operation.
