# Supplementary material for: Effect of Ileal Transposition (IT) on Angiopoietin-Like Protein-8 (ANGPTL8) and Pentraxin (PTX3) Plasma Level in Sprague-Dawley Rats Fed High-Fat Diet (HFD)
Source: Int J Endocrinol. 2021 May 5;2021:6699923. doi: 10.1155/2021/6699923 (PMC8118740; doi:10.1155/2021/6699923)
Supplement: Supplementary Materials — Table 1: Median (M) and quartile deviation (Q25 and Q75; 25th and 75th percentiles, respectively) of ANGPTL8 plasma concentration in four diet groups according to operation type. ∗ or # denotes significant difference between median values; Kruskal–Wallis test, p < 0.05 (group size: n = 6, except IT HFD/HFD: n = 5). IT—ileal transposition, SHAM—sham operated animals, CD—control diet, and HFD—high-fat diet. Preoperative diet/postoperative diet: CD/HFD, CD/CD, HFD/HFD, HFD/CD. Table 2: Median (M) and quartile deviation (Q25 and Q75; 25th and 75th percentiles, respectively) of PTX3 plasma concentration in four diet groups according to operation type. ∗ denotes significant difference between median values; Kruskal–Wallis test, p < 0.05 (group size: n = 6, except IT HFD/HFD: n = 5).IT—ileal transposition, SHAM—sham operated animals, CD—control diet, and HFD—high-fat diet. Preoperative diet/postoperative diet: CD/HFD, CD/CD, HFD/HFD, HFD/CD. Table 3: Median (M) and quartile deviation (Q25 and Q75; 25th and 75th percentiles, respectively) of leptin plasma concentration in four diet groups according to operation type. ∗ or # denotes significant difference between median values; Kruskal–Wallis test, p < 0.05(group size: n = 6, except IT HFD/HFD: n = 5). IT—ileal transposition, SHAM—sham operated animals, CD—control diet, and HFD—high-fat diet. Preoperative diet/postoperative diet: CD/HFD, CD/CD, HFD/HFD, HFD/CD. Table 4. Median (M) and quartile deviation (Q25 and Q75; 25th and 75th percentiles, respectively) of adiponectin plasma concentration in four diet groups according to operation type. ∗ or # denotes significant difference between median values; Kruskal–Wallis test, p < 0.05(group size: n = 6, except IT HFD/HFD: n = 5).IT—ileal transposition, SHAM—sham operated animals, CD—control diet, and HFD—high-fat diet. Preoperative diet/postoperative diet: CD/HFD, CD/CD, HFD/HFD, HFD/CD. Supplement. Fig. 1: Postoperatively glucose tolerance profile. (A) Area under curve of blood [file 6699923.f1.docx]

Supplementary data

**Table 1.** Median (M) and quartile deviation (Q25 and Q75; 25th and 75th percentiles respectively) of **ANGPTL8** plasma concentration in four diet groups according to operation type. Sign * or ^#^ denotes significant difference between median values; Kruskal–Wallis test, p < 0.05 (group size: n=6, except IT HFD/HFD: n=5). Abbreviations: IT- ileal transposition, SHAM- sham operated animals. CD-control diet, HFD- high fat diet. Preoperative diet/postoperative diet: CD/HFD, CD/CD, HFD/HFD, HFD/CD

| **ANGPTL8**  [pg/mL] | DIET  PRE/POST OP | M | Q25 | Q75 | DIET  PRE/POST OP | M | Q25 | Q75 |
| --- | --- | --- | --- | --- | --- | --- | --- | --- |
| IT | CD/HFD | **377.8*** | 232.9 | 668.7 | HFD/HFD | **1751.2 *** | 1383.7 | 2222.0 |
|  | CD/CD | **900.3*** | 899.0 | 909.5 | HFD/CD | **2301.2^#^** | 1722.2 | 3042.9 |
| SHAM | CD/HFD | 912.9 | 582.9 | 1856.2 | HFD/HFD | **594.5*** | 447.9 | 690.5 |
|  | CD/CD | 822.9 | 245.4 | 2072.0 | HFD/CD | **922.9^#^** | 626.2 | 1530.8 |

**Table 2.** Median (M) and quartile deviation (Q25 and Q75; 25th and 75th percentiles respectively) of **PTX3** plasma concentration in four diet groups according to operation type. Sign * denotes significant difference between median values; Kruskal–Wallis test, p < 0.05 (group size: n=6, except IT HFD/HFD: n=5). Abbreviations: IT- ileal transposition, SHAM- sham operated animals. CD-control diet, HFD- high fat diet. Preoperative diet/postoperative diet: CD/HFD, CD/CD, HFD/HFD, HFD/CD

| **PTX3**  [pg/mL] | DIET  PRE/POST OP | M | Q25 | Q75 | DIET  PRE/POST OP | M | Q25 | Q75 |
| --- | --- | --- | --- | --- | --- | --- | --- | --- |
| IT | CD/HFD | **2098.7*** | 1554.5 | 2778.3 | HFD/HFD | 1489.4 | 1100.5 | 2583.8 |
|  | CD/CD | **710.6*** | 678.5 | 800.5 | HFD/CD | 1945.0 | 1661.6 | 2028.3 |
| SHAM | CD/HFD | 1711.6 | 1428.3 | 2461.6 | HFD/HFD | 2167.2 | 2100.5 | 2322.7 |
|  | CD/CD | 1428.3 | 1097.7 | 1953.3 | HFD/CD | 2011.6 | 1167.2 | 2833.8 |

**Table 3.** Median (M) and quartile deviation (Q25 and Q75; 25th and 75th percentiles respectively) of **LEPTIN** plasma concentration in four diet groups according to operation type. Sign * or ^#^ denotes significant difference between median values; Kruskal–Wallis test, p < 0.05(group size: n=6, except IT HFD/HFD: n=5). Abbreviations: IT- ileal transposition, SHAM- sham operated animals. CD-control diet, HFD- high fat diet. Preoperative diet/postoperative diet: CD/HFD, CD/CD, HFD/HFD, HFD/CD

| **Leptin**  [ng/mL] | DIET  PRE/POST OP | M | Q25 | Q75 | DIET  PRE/POST OP | M | Q25 | Q75 |
| --- | --- | --- | --- | --- | --- | --- | --- | --- |
| IT | CD/HFD | **245.7*** | 209.6 | 269.4 | HFD/HFD | 274.5 | 202.5 | 339.9 |
|  | CD/CD | **107.8*** | 94.6 | 189.4 | HFD/CD | 215.4 | 177.7 | 277.2 |
| SHAM | CD/HFD | 126.1 | 119.4 | 213.0 | HFD/HFD | 214.1 | 182.2 | 339.9 |
|  | CD/CD | 179.6 | 98.3 | 201.0 | HFD/CD | 193.5 | 159.7 | 353.7 |

**Table 4.** Median (M) and quartile deviation (Q25 and Q75; 25th and 75th percentiles respectively) of **ADIPONECTIN** plasma concentration in four diet groups according to operation type. Sign * or ^#^ denotes significant difference between median values; Kruskal–Wallis test, p < 0.05(group size: n=6, except IT HFD/HFD: n=5). Abbreviations: IT- ileal transposition, SHAM- sham operated animals. CD-control diet, HFD- high fat diet. Preoperative diet/postoperative diet: CD/HFD, CD/CD, HFD/HFD, HFD/CD

| **Adiponectin**  [ng/mL] | DIET  PRE/POST OP | M | Q25 | Q75 | DIET  PRE/POST OP | M | Q25 | Q75 |
| --- | --- | --- | --- | --- | --- | --- | --- | --- |
| IT | CD/HFD | **43.7*** | 32.3 | 61.7 | HFD/HFD | 72.6 | 68.4 | 86.4 |
|  | CD/CD | **32.1*** | 9.6 | 44.5 | HFD/CD | 42.4 | 32.9 | 56.9 |
| SHAM | CD/HFD | 29.4 | 14.7 | 54.0 | HFD/HFD | 28.7 | 26.3 | 31.1 |
|  | CD/CD | 11.9 | 7.3 | 21.9 | HFD/CD | 38.3 | 21.1 | 49.5 |

Sawczyn et.al. 2019, Peptides

**Supplement. Fig.1**

Postoperatively glucose tolerance profile.

**(A)** Area Under Curve of blood glucose profiles of OGTT. *denotes significant difference

between SHAM CD/CD and SHAM CD/HFD groups. # denotes significant difference

between SHAM HFD/HFD and SHAM CD/HFD

**(B)** OGTT curves in four groups of pre/postoperative diet according to the operation type IT or

SHAM surgery

* denotes significant difference between fasting state IT CD/CD and SHAM CD/CD

# denotes significant difference between IT CD/HFD and SHAM CD/HFD, 30 min of OGTT

† denotes significant difference between IT CD/HFD and SHAM CD/HFD, 60 min of OGTT

‡ denotes significant difference between SHAM HFD/HFD and SHAM CD/HFD

denotes significant difference between groups. p<0.05 by Tuckey’s test. Preoperative

diet/postoperative diet: CD/HFD, CD/CD, HFD/HFD, HFD/CD. Data are expressed as means ±SD, n=6. p<0.05 by Tuckey’s test

Sawczyn et.al. 2019, Peptides

**Supplement. Fig. 2**

Weight changes during the experiment.

**(A)** Weight after 8 weeks of HFD and CD, * denotes significant difference between groups

**(B)** Variability of weight fluctuations in five time periods after the operation, * denotes

significant difference between IT CD/CD and SHAM CD/CD groups; # denotes significant

difference between SHAM CD/CD and SHAM CD/HFD groups; † denotes significant

difference between IT HFD/HFD and SHAM HFD/HFD groups

**(C)** Body weights changes during 8 weeks after operation.
